# Investigating Rheological Behavior of *Chlorella vulgaris* Starch: Implications for 3D Printable Bioplastic Material

**DOI:** 10.3390/polym18121452

**Published:** 2026-06-10

**Authors:** Kokeb Hurruma Jiru, Hirpa G. Lemu, Eyosias Tamerat, Mesay Tolcha

**Affiliations:** 1 Faculty of Mechanical Engineering, Jimma Institute of Technology, Jimma University, Jimma P.O. Box 378, Ethiopia; 2 Department of Mechanical and Structural Engineering, University of Stavanger, P.O. Box 8600, 4036 Stavanger, Norway; hirpa.g.lemu@uis.no; 3 Bio and Emerging Technology Institute, Addis Ababa P.O. Box 5954, Ethiopia; eyosias722@gmail.com

**Keywords:** *C. vulgaris*, starch, viscoelastic, rheology, bioplastic, 3D printing, DIW

## Abstract

The increasing demand for sustainable materials in additive manufacturing has driven the development of bioplastics derived from renewable biomass, including microalgae. In this study, the rheological behavior of a 20 wt.% aqueous gel prepared from native *Chlorella vulgaris (C. vulgaris)* starch, plasticized with 30 wt.% glycerol, was investigated to assess its suitability for extrusion-based 3D printing (direct-ink-writing, DIW). Steady shear analysis revealed a pronounced yield stress ($\tau_{0}$ = 271.93 Pa) and strong shear-thinning behavior, described by the Herschel–Bulkley model (*K* = 59.47 Pa·s^n^, *n* = 0.67), indicating structural stability at rest and efficient flow under shear. Oscillatory measurements confirmed a predominantly elastic response, with storage modulus (G′ ≈ 13,500 Pa) greatly exceeding loss modulus (G″) and a low loss factor (tan δ≈ 0.1), demonstrating gel integrity and shape retention. Temperature-dependent analysis indicated enhanced network strength without thermal softening, while thixotropic recovery tests showed rapid structural rebuilding after shear removal. Notably, a ~50% increase in G′ during recovery highlights improved interlayer adhesion potential. These results show that *C. vulgaris* starch exhibits the key rheological characteristics required for DIW-type extrusion printing, including yield stress, shear-thinning behavior, viscoelastic stability, and rapid recovery, making it a promising candidate for this application.

## 1. Introduction

Additive manufacturing, commonly referred to as 3D printing, represents a revolutionary technology in the fields of materials science and manufacturing engineering. It facilitates the creation of highly complex geometries, including those with internal voids, in a single process without the need for multiple assembly steps or molds. Operating on the principle of precise, layer-by-layer deposition guided by digital designs from computer-aided design (CAD) software. It provides unparalleled design freedom and rapid prototyping capabilities [1,2]. A key factor that further elevates the value of this technology is the growing ability to employ sustainable raw materials that combine good printability with meaningful environmental benefits [3]. This synergy has driven a significant shift toward eco-friendly, biodegradable feedstocks that support high-performance manufacturing while reducing environmental impact [4]. For example, biopolymers derived from renewable resources such as plants or microbial fermentation have gained considerable attention as green alternatives to conventional 3D printing materials. A notable case is polylactic acid (PLA), a widely used biodegradable polymer produced from starch [5].

Starch is the most abundant, lowest-cost, and fully biodegradable material that can be converted into thermoplastic starch for processing as a 3D-printable feedstock [6]. It originates from a wide range of plant sources, including corn, potato, cassava, wheat, rice, sugarcane, and even algae, each imparting distinct rheological characteristics that strongly influence extrusion behavior. These differences arise from variations in amylose-to-amylopectin ratio [7,8], crystallinity, granule morphology, chain length, and lipid and protein content, all of which govern molecular interactions, swelling behavior, and network formation in water, thereby affecting viscosity, gel strength, and retrogradation tendencies [9]. For example, starches with high amylose content exhibit markedly increased viscosity and enhanced gel stability, properties that help maintain structural integrity under shear and compression forces during material deposition in extrusion-based 3D printing. This quantitative relationship is demonstrated by Xie et al. [10], who studied corn starches with amylose contents ranging from 0% to 80%. They reported that the apparent viscosity at a shear rate of 100 s^−1^ increased from 277 Pa·s at 0% amylose to 1254 Pa·s at 80% amylose, confirming that amylose substantially contributes to flow resistance. Similar trends were observed by Qiao et al. [11], further validating the strong influence of amylose content on starch rheology.

Crystallinity is another important factor influencing starch rheology. High-crystallinity starches exhibit higher yield stress, providing structural stability during cooling of printed constructs, whereas low-crystallinity starches promote higher flowability, an important requirement for continuous extrusion. Velazquez et al. [12] corroborated these findings in corn starch, showing that long-term retrogradation increases gel crystallinity, thereby enhancing G′, gel strength, and elastic behavior, albeit at the cost of a reduced network mesh size. These post-printing changes indicate that printed parts may gain stiffness and dimensional stability during aging. Regarding residual protein, Lin et al. [13] demonstrated that adding protein to Indica and Japonica rice starch decreases apparent viscosity, consistency coefficient, and yield stress, while increasing G′. This indicates a balance between lower flow resistance and mechanical robustness, both of which play a critical role in determining printability and ensuring that the material preserves its shape during extrusion-based 3D printing.

Although the rheological properties of starches derived from various terrestrial plant sources have been extensively characterized, including in the context of 3D printing applications, the rheological behavior of microalgal starches remains markedly understudied. This gap is notable because microalgal starch differs fundamentally from plant-based starches in its biological origin, granule architecture, and environmental adaptability [14,15]. Microalgae typically produce smaller and more uniformly sized granules, and their starch biosynthesis pathways respond strongly to stress conditions such as nitrogen starvation, which can substantially increase total starch accumulation and alter carbon partitioning [16,17]. While current evidence does not conclusively show modulation of the amylose content or crystallinity under such stresses, the distinct granule architecture and stress-induced metabolic shifts may still yield starches with unique rheological and gelation behaviors relevant to extrusion-based additive manufacturing. Moreover, microalgae can be cultivated rapidly, year-round, and independently of arable land, offering a sustainable and scalable alternative to crop derived starches [18,19].

Another key advantage of microalgae over conventional plant sources is their capacity to be deliberately manipulated to enhance starch production beyond natural baseline levels. This is achieved by exposing cells to controlled stress conditions that redirect cellular metabolism toward starch accumulation as a survival strategy [20]. Such stresses typically involve limiting essential nutrients such as nitrogen, phosphorus, or sulfur, or applying environmental cues including red-light exposure or elevated temperatures [21,22,23]. Under these conditions, microalgae suppress growth-associated pathways and channel carbon toward storage compounds, particularly starch. Numerous studies have demonstrated that this approach can induce microalgae to accumulate exceptionally high starch levels, in some cases reaching up to 60% of the biomass on a dry-weight basis [24], providing a reliable and tunable feedstock supply for 3D printing.

Within this broader group of starch-accumulating microalgae, *Chlorella vulgaris* (*C. vulgaris*) has emerged as one of the most practical and industrially relevant candidates. Its robustness, adaptability to fluctuating environments, and strong physiological response to stress enable it to consistently achieve high starch contents [25]. Equally important, *C. vulgaris* exhibits high biomass productivity, with reported growth rates of 2.0–3.5 g L^−1^ day^−1^ under optimized phototrophic conditions and >1 g L^−1^ day^−1^ in heterotrophic cultivation [26,27]. This combination of rapid growth and strong starch inducing stress responses makes the microalga a particularly efficient biological platform for starch production. Supporting its technological relevance, Six et al. [28] successfully extracted starch from this alga and processed it into thermoplastic starch via extrusion, demonstrating that the starch can exhibit more ductile behavior than conventional plant based starches, an attribute highly advantageous for extrusion based bioplastic and 3D printing applications. In addition, the starch of *Chlorella* is characterized by an ultra-small granule size, typically less than 1.5 μm in diameter, which promotes improved flow during extrusion and enhances the interaction between starch and plasticizers [15]. Its semi crystalline A type structure and high surface area further contribute to efficient thermal–mechanical processing [29]. Together, these attributes provide a strong scientific basis for the suitability of *Chlorella* starch in extrusion driven bioplastic and emerging 3D printing applications.

Despite these promising characteristics, the rheological properties of *C. vulgaris* starch have not yet been scientifically characterized, which makes it difficult to predict its performance in extrusion-based 3D printing. To the best of our knowledge, the only systematic investigations on the rheology of microalgal starch are those conducted by Jiang et al. [30,31]. Their work examined the steady shear and frequency dependent behavior of starch extracted from several microalgal species, including *C. sorokiniana*, *C. ellipsoidea*, *C. luteorividis*, *C. protothecoides*, and *C. pyrenoidosa*. Across these studies, they consistently reported pronounced shear thinning behavior that compared well with starches from rice, wheat, and corn. However, several critical rheological parameters essential for evaluating 3D printability, particularly temperature-dependent viscoelastic transitions and thixotropic recovery mechanisms, have not yet been systematically addressed in the literature.

To address this knowledge gap, the present study provides a comprehensive rheological characterization of starch extracted from a native Ethiopian strain of *C. vulgaris*. The analysis encompasses steady-shear flow behavior as well as dynamic viscoelastic properties, including frequency, temperature, and time dependence. It was hypothesized that the starch from Ethiopian *C. vulgaris* would exhibit significant thixotropic recovery and favorable viscoelastic characteristics, thereby indicating strong potential for application in extrusion-based 3D printing, specifically DIW.

## 2. Materials and Methods

### 2.1. Materials and Sample Preparation

Native starch isolated from *Chlorella vulgaris* (*C. vulgaris*) microalga using ultrasonic homogenization in DMSO (99.7%) followed by cold ethanol (96%) precipitation was used in this investigation, with the homogenization performed for 30 min in pulsation mode (1 s on, 0.5 s off) at 97% power output. The extracted starch contained 20.74% amylose and 79.26% amylopectin, with an average granule size of approximately 1.12 μm and a moisture content of ~13% (*w*/*w*). Cosmetic-grade glycerol (100% purity; Theraderm, Ethiopia) served as the plasticizer. Prior to formulation, the starch was freeze-dried for 24 h in a lyophilizer (Alpha 2–4 LDplus, CHRIST, Osterode am Harz, Germany) and subsequently dispersed in distilled water at 20 wt.%, a concentration selected based on preliminary trials showing that lower starch contents yielded gels with insufficient strength for shape retention, whereas higher concentrations produced excessive viscosity and poor flow behavior. The dispersion was gently stirred for 20 min to ensure uniform mixing, then gelatinized by heating at 95 °C for 20 min with the beaker covered to minimize moisture loss. Immediately after gelatinization, glycerol was added at 30 wt.% relative to starch and mixed thoroughly, and the resulting formulation was allowed to cool to room temperature before testing.

### 2.2. Rheological Measurements

Rheological characterization was performed using a rotational rheometer (MCR 102, Anton Paar, Graz, Austria) equipped with a 25 mm parallel-plate geometry and a fixed 1 mm gap. Samples were carefully loaded onto the lower plate, trimmed to remove excess material, and allowed to equilibrate for 2 min prior to measurement. To minimize moisture loss during testing, a thin layer of low-density silicone oil was applied around the sample perimeter. All measurements were conducted at 25 °C unless otherwise specified, with temperature controlled by a Peltier system to ensure thermal stability throughout the experiments. Before each steady-shear test, the sample was subjected to a pre-conditioning step consisting of shear at 1 s^−1^ for 60 s followed by a 60 s rest period to ensure a reproducible structural state for this thixotropic starch gel. Steady-shear flow behavior was then evaluated using RheoCompass software version 2.3.2 by applying a logarithmic shear-rate ramp from 0.01 to 40 s^−1^ over 12 s, with the upward ramp used for model fitting to avoid artifacts from thixotropic recovery. The Herschel–Bulkley model was selected because starch gels typically behave as yield-stress, shear-thinning fluids, assuming a finite yield stress prior to flow initiation, a power-law relationship between shear stress and shear rate after yielding, and negligible wall slip under the chosen geometry. Model parameters ($\tau_{0}$, *K*, *n*) were obtained using nonlinear regression in Rheology Lite software version 1.1.4.0, and goodness-of-fit was assessed using R^2^ values and residual analysis to ensure reliable representation of the experimental data.

Dynamic shear measurements were performed to characterize the viscoelastic behavior of the gelatinized starch. The linear viscoelastic region (LVE) was first identified through an amplitude sweep from 0.01% to 1000% strain at 1 Hz (6.28 rad/s) over 300 s, and all subsequent oscillatory tests were conducted at 0.5% strain, well within the LVE. Frequency sweep tests were carried out from 0.01 to 100 Hz (0.06–628.3 rad/s) to determine storage modulus (G′), loss modulus (G″), tan δ, and complex viscosity (η*). Temperature-dependent viscoelastic behavior was assessed using a temperature sweep from 25 to 95 °C at a heating rate of 2 °C/min under continuous oscillation at 1 Hz (6.28 rad/s). Structural stability over time was evaluated using a 20 min time sweep at 0.5% strain and 1 Hz (6.28 rad/s). Thixotropic breakdown and recovery behavior were examined using a three-interval thixotropy test (3ITT), consisting of pre-shear at 1 s^−1^, followed by low-shear (0.5 s^−1^ for 120 s), high-shear (300 s^−1^ for 60 s), and final low-shear (0.5 s^−1^ for 600 s) intervals.

All rheological measurements were performed in triplicate, and the resulting datasets were statistically evaluated in OriginPro version 10.3. For each parameter, the mean, standard deviation (SD), standard error of the mean (SE of mean), and coefficient of variation (CV) were calculated to assess central tendency and relative variability. Where applicable, 95% confidence intervals (CI) were computed to quantify the precision of the estimated mean values. Curve-fitting procedures, residual analysis, and graphical outputs were also generated using OriginPro version 10.3 to ensure consistent and reproducible data processing.

### 2.3. Scanning Electron Microscope

To examine the internal structures of the starch gel without inducing plastic deformation, the sample was quenched in liquid nitrogen for 10 min. The frozen sample was then quickly extracted and fractured with a pre-chilled blade to expose a clean cross-section. The fractured pieces were immediately mounted onto aluminum stubs using double-sided carbon tape and coated with a thin layer of silver via a sputter coater (Leica EM ACE 600, Leica Microsystems, Vienna, Austria). Imaging was performed using a scanning electron microscope (SEM) (JSM-IT800, JEOL, Akishima, Tokyo, Japan) at an accelerating voltage of 10 kV under high vacuum conditions.

## 3. Results and Discussion

### 3.1. Flow Behavior

**Figure 1:**
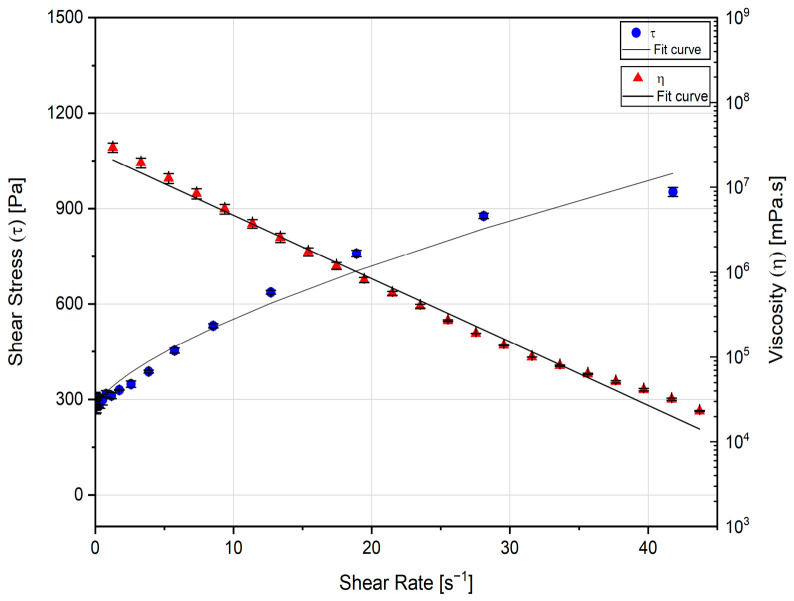
Shear rate versus shear stress and apparent viscosity for Chlorella vulgaris (*C. vulgaris*) starch (20 wt.%). Data points represent the mean ± SD of triplicate measurements (*n* = 3).

The shear rate ($\dot{\gamma }$) versus shear stress (τ) and apparent viscosity (η) data for *Chlorella vulgaris* (*C. vulgaris*) starch, illustrated in Figure 1, shows pseudoplastic (shear-thinning) behavior. This behavior is well fitted by the Herschel–Bulkley model (R^2^ = 0.982), expressed as:

(1) $$\tau =\tau_{0}+{K\dot{\gamma }}^{n}$$

**Figure 2:**
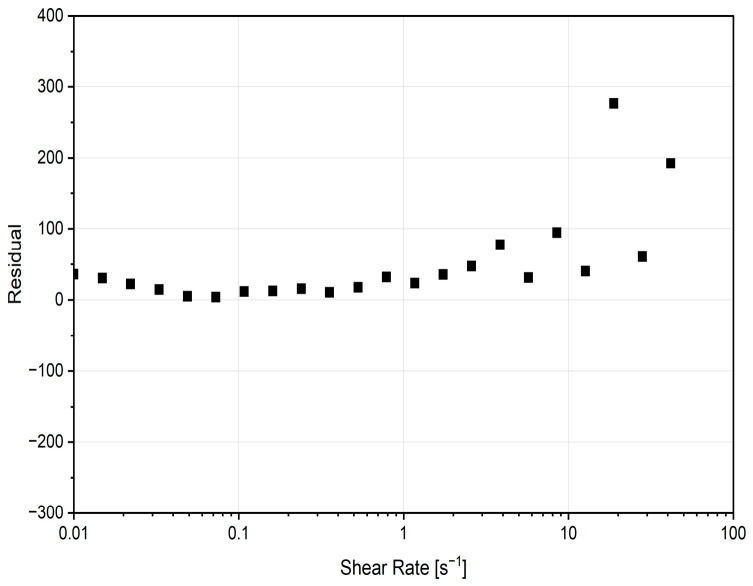
Residuals from the Herschel–Bulkley fit to shear stress data for *C. vulgaris* starch (20 wt.%).

Non-linear regression analysis of the model gives a yield stress ($\tau_{0}$) of 271.93 Pa, a consistency index (*K*) of 59.47 Pa·s^n^, and a flow behavior index (*n*) of 0.67. These values indicate a potentially favorable balance of properties for DIW-type extrusion-based 3D printing. The measured yield stress likely supports the material’s ability to retain its shape after deposition, while the moderate consistency index and pronounced shear-thinning behavior (*n* < 1) imply efficient viscosity reduction under nozzle shear. Together, these characteristics enable smooth extrusion and rapid structural recovery, thereby enhancing interlayer adhesion. The adequacy of the model is further supported by the residual analysis shown in Figure 2, which exhibits predominantly random scatter around zero. The slight positive deviations at high shear rates suggest minor underprediction of stress, likely associated with shear-induced structural breakdown. Compared with botanical starches such as corn and mashed potato (Table 1), which exhibit substantially higher yield stresses (≈800 Pa and 312.16 Pa, respectively) and may require elevated extrusion pressures with increased clogging risk, *C. vulgaris* starch demonstrates a moderate yield stress that lies well within the reported successful printing window of 32–455 Pa for corn, rice, and potato starches [32]. Its relatively low *K*, higher *n*, ultra-small granule size (1.12 μm), and high amylopectin content (79.26%) collectively promote improved dispersion, uniform gelatinization, reduced agglomeration, and enhanced interlayer adhesion. The apparent viscosity at 1 s^−1^ (≈400 Pa·s), markedly lower than that of rice, potato, or buckwheat starches, further supports efficient extrusion through fine nozzles while maintaining sufficient structural stability during deposition. This moderate viscosity, together with the observed pseudoplastic behavior, may facilitate effortless extrusion through fine nozzles by enabling significant viscosity reduction at shear rates between 0.01 and 40 s^−1^.

The reliability of the rheological interpretations is supported by the consistency of the underlying measurements. Across the measured shear-rate range, SE remained small relative to the mean, and CVs were consistently <8% for shear stress and <13% for viscosity. In addition, 95% CI narrowed progressively at higher shear rates, confirming excellent reproducibility and strengthening confidence in the flowability assessment.

### 3.2. Dynamic Shear Properties

#### 3.2.1. Amplitude Sweep

**Figure 3:**
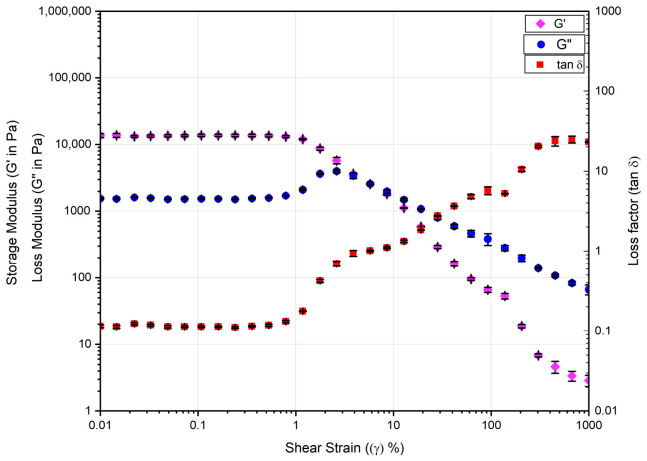
Amplitude sweep dependency of G′, G″, and tan δ for *C. vulgaris* starch (20 wt.%). Data points represent the mean ± SD of triplicate measurements (*n* = 3).

The critical strain (${\dot{\gamma }}_{c}$) at which the properties of starch no longer remain constant under deformation was determined using the identification of the limit of the linear viscoelastic region (LVE). It can be seen from Figure 3 that in the strain range from 0.01 to 0.6%, the average G′ of the material was around 13,500 Pa, while G″ was about 1500 Pa, leading to a low tan δ of around 0.1, indicating that the gel structure is strong and rigid when it is under small deformation. Beyond this critical strain of about 0.6%, the G′ starts a gradual decrease, followed by a sharper drop, while G″ simultaneously increases, causing tan δ to increase sharply. Such strain-stiffening behavior reflects the progressive mobilization and alignment of the network elements under moderate deformations that lead to temporary enhancement of viscous dissipation before its irreversible breakdown. At the crossover point, around 4% of strain, G′ = G″ approximately 3400–3600 Pa, which indicates the transition to viscous dominance where the paste starts behaving much more like a fluid. Beyond this crossover point, G′ and G″ decrease considerably. For example, around 100% of strain, they drop to 66 and 383 Pa, respectively, while at 1000% of strain, they reach 3 and 67 Pa, respectively. Concurrently, tan δ exceeds 1 and eventually reaches values above 10, confirming complete yielding and flow behavior characteristic of a thick liquid that is an important indicator of flowability under large deformation.

The amplitude-sweep measurements also demonstrated strong statistical consistency. SE of the mean remained small relative to G′ and G″ across the strain range, even near the onset of nonlinearity. CVs were low in the LVE region, typically 0.4–11% for G′ and 0.7–10% for G″, indicating tightly clustered replicates and stable material response. The 95% CIs for both moduli were narrow at low strains and widened progressively at higher strains, consistent with the transition from a well-defined elastic network to a more dissipative regime.

#### 3.2.2. Frequency Sweep

**Figure 4:**
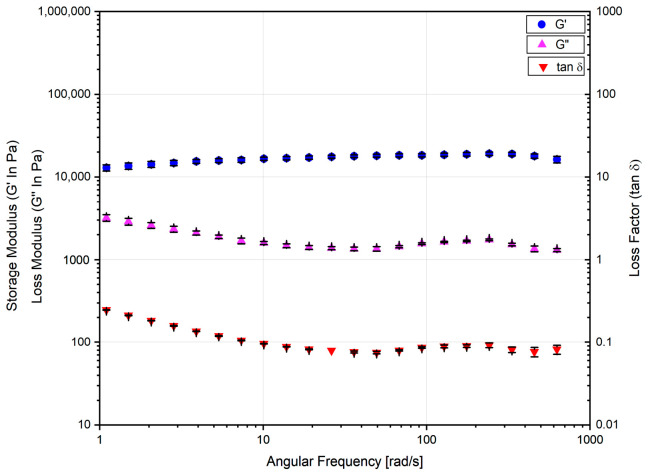
Frequency sweep dependency of G′, G″, and tan δ for *C. vulgaris* starch (20 wt.%). Data points represent the mean ± SD of triplicate measurements (*n* = 3).

A dynamic frequency sweep test was done in the identified LVE region at 0.5% strain to investigate the dependency of elastic and viscous moduli on frequency. Figure 4 shows that the G′ remains constant over a large range of frequencies, underlining that starch has a strong and well-structured elastic network. This tells the material’s ability to support its structural strength during the extrusion-cooling process without sagging or collapse, facilitating effective layering throughout the process of DIW [37,38]. Additionally, the dominantly elastic nature of starch, shown by G′ > G″ and tan δ < 1, indicates minimal energy losses during deformation, supported by the slight decrease in G″ and tan δ with frequency, where dissipation remains lower than stored energy. This observation is also important in DIW because an excessive viscous nature in materials results in undesirable flow during the printing process. In general, the material’s stability over various frequencies implies its adaptiveness to a wide range of printing speeds without an adverse effect on the quality of the prints.

This observation is reinforced by the statistical analysis of the frequency-sweep data, which showed high measurement consistency across the full angular-frequency domain. For G′, SE of the mean remained small relative to the modulus values, ranging from ~413 to 872 Pa even as G′ increased from ~12,880 Pa at 1.1 rad s^−1^ to ~18,245 Pa at 93.5 rad s^−1^. CVs were low, typically 4–9%, indicating tightly clustered replicates and a stable elastic response. G″ showed similarly strong reproducibility, with SE values generally between 16 and 168 Pa and CV values mostly 2–9%. The 95% CIs for both moduli were narrow at all frequencies.

#### 3.2.3. Temperature Sweep

**Figure 5:**
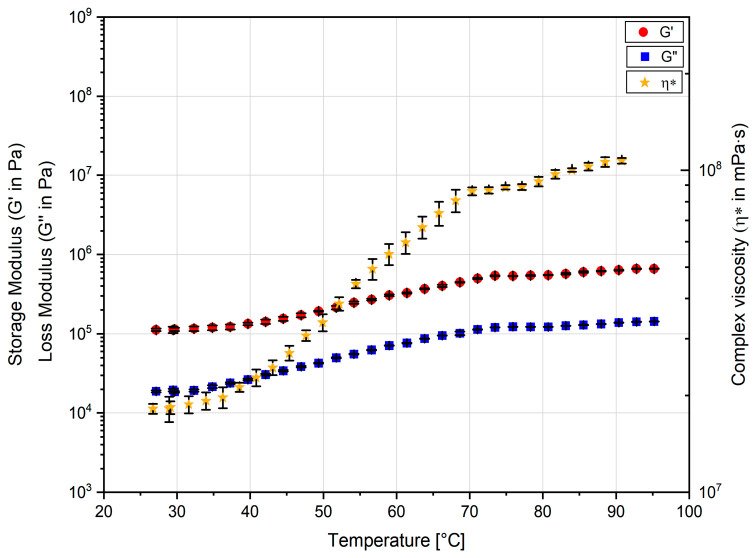
Temperature sweep dependency of G′, G″, and η* for *C. vulgaris* starch (20 wt.%). Data points represent the mean ± SD of triplicate measurements (*n* = 3).

The temperature sweep rheological analysis of *C. vulgaris* starch confirms the predominantly elastic viscoelastic behavior observed in the amplitude and frequency sweep experiments. As shown in Figure 5, the G′ remains consistently higher than the G″ across the entire temperature range, indicating solid-like behavior with no crossover point where viscous dominance would occur. Compared to conventional botanical starches, *C. vulgaris* starch exhibits superior temperature-dependent rheological properties for 3D printing applications. Its gelation temperature of 92.7 °C is notably higher than the 60.5–79 °C range reported for potato, corn, rice, tapioca, pea, mung bean and other starches (Table 2). This elevated gelation temperature possibly provides a wider and more forgiving extrusion window, reducing the risk of premature gelation and nozzle clogging during printing. Most importantly, the maximum G′ of *C. vulgaris* starch reaches 6.6 × 10^5^ Pa, approximately 40 to 600 times higher than the typical values (1000–15,400 Pa) observed for other starches. This exceptional gel strength likely enables excellent shape fidelity, allowing the fabrication of tall, complex, and self-supporting structures with minimal deformation. The tan δ value at G′max (0.21) lies comfortably within the range of other starches (0.06–0.29), reflecting a well-balanced viscoelastic character that supports both smooth extrusion and structural integrity.

The complex viscosity (η*) profile further supports these findings. η* increases gradually with temperature, starting from 1.8 × 10^7^ mPa·s at 25–30 °C and rising monotonically until approximately 9 °C, after which it plateaus at around 1.06 × 10^8^ mPa·s. This trend aligns well with the observed increases in G′ and G″, confirming the absence of thermal softening. Instead, the material exhibits thermally induced viscosity build-up, indicative of enhanced molecular entanglement and stable gel network formation without structural breakdown. These characteristics highlight the outstanding thermal stability and viscoelastic strength of *C. vulgaris* starch under elevated temperatures.

Such differences in behavior can be reasonably attributed to intrinsic compositional and microstructural features of the starch. A higher amylose-to-amylopectin ratio or more compact granule morphology may contribute to the enhanced thermal resistance observed. Additionally, although not experimentally verified in this study, weak associations with residual cellular proteins or lipids could hypothetically influence network reinforcement, as has been suggested for some non-terrestrial starches [39,40]. These factors may collectively promote strengthening of the gel network rather than its degradation at elevated temperatures. These features have direct implications for 3D printability: the elastic dominance of the material (G′ > G″) supports high shape fidelity, while the temperature-dependent increase and subsequent stabilization of complex viscosity help maintain structural integrity during deposition. Together with the preservation of network structure post-extrusion and controlled flow within the nozzle, these characteristics likely facilitate smooth layer-by-layer deposition with minimal slumping.

The statistical behavior of G′, G″, and complex viscosity across the temperature sweep showed clear and quantifiable trends that reflect the thermal evolution of the starch network. For G′, the SE of the mean ranged from approximately 1947 to 7251 Pa, representing only 0.8–3.5% of the corresponding mean values. Even at higher temperatures, where G′ increased sharply toward ~600,000 Pa, the SE remained proportionally small, typically 3200–7200 Pa, maintaining a CV below 10% for most of the dataset. G″ exhibited similarly stable statistics: SE values generally fell between 242 and 570 Pa, with CV values commonly 0.3–3%, indicating tight clustering of replicates. Complex viscosity followed the same pattern, with CV values mostly 1–9%. The 95% confidence intervals for all three parameters remained narrow across the full temperature range and expanded only slightly at the highest temperatures, reflecting increased thermal mobility.

**Table 2:** Temperature sweep parameters for 20 wt.% starch suspensions for *C. vulgaris* starch vs. various botanical sources.

Starch	T_G′max_ (°C)	G′_max_ (Pa)	Tan δ_G′max_	Ref.
Potato	68.4	2546	0.26	[32]
Corn	69.5	1655	0.25	
Rice	76.8	6122	0.08	
Potato	60.5	2595.5	0.24	[41]
Tapioca	62.1	995.6	0.29	
Waxy corn	63.6	1450	0.19	
Sweet potato (TNu17)	63.6	4735.5	0.11	
Sweet potato (TNu57)	69.8	3447.5	0.20	
High amylose corn	79.1	8879.5	0.06	
Corn	65.2	6365	0.12	
Rice (TNu67)	62.1	10,321.5	0.07	
Pea	69.8	15,400	0.12	
Mung bean	66.7	9853	0.14	
Potato	69.8	2670	0.27	[42]
Waxy potato	72.0	1100	0.28	
Cashew kernel	75.0	5724.53	0.19	[43]
*C. vulgaris*	92.7	6.6 × 10^5^	0.21	This study

#### 3.2.4. Three-Interval Thixotropic Test

**Figure 6:**
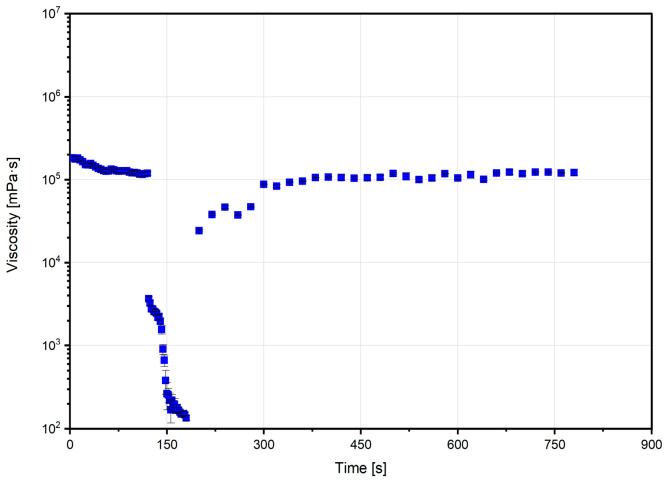
Viscosity recovery behavior of *C. vulgaris* starch (20 wt.%). Data points represent the mean ± SD of triplicate measurements (*n* = 3).

The thixotropic behavior of *C. vulgaris* starch, depicted in Figure 6, reveals the starch’s good recovery capacity after shear. At low shear (0.5 s^−1^ for 60 s), *C. vulgaris* starch gel exhibits a constant resting viscosity around 10^5^ mPa.s, indicating a well-structured gel state. When high shear is applied (300 s^−1^ for 120 s, which is a simulation of extrusion through a nozzle), viscosity drops sharply by over six orders of magnitude to below 100 mPa.s, showing pronounced shear thinning and good flowability under printing conditions. After removal of shear, 0.5 s^−1^ recovery phase for 600 s, viscosity recovers after a 20 s delay, with a recovery of 102%, representing almost complete recovery. This near-instantaneous and complete rebuild of the three-dimensional network may be driven by rapid reformation of hydrogen bonds, ionic interactions, and physical entanglements among retrograded starch chains and algal biopolymers [44]. This confirms outstanding thixotropic recovery and ensures that the gel flows smoothly during printing but immediately regains rigidity upon deposition to prevent slumping, enable sharp features, and promote strong interlayer fusion.

Across all 3-ITT stages, SE of mean values remained small relative to the mean, with CV generally <6% during the initial low-shear stage, <36% immediately after high-shear breakdown, where the transiently low viscosity naturally amplifies relative variability, and <10% throughout the recovery period. The corresponding 95% confidence intervals remained reasonably narrow, confirming reproducible thixotropic breakdown and structural rebuilding.

The results of the 3ITT are further supported by the time sweep results (Supplementary Materials), which depict a strongly elastic gel undergoing continuous, balanced network reinforcement over 20 min. The G′ increases steadily from 16,046 Pa at t = 0 min to 23,998 Pa at t = 20 min (≈50% rise), reflecting the progressive formation of a more rigid three-dimensional network, possibly driven by amylose retrogradation, hydrogen bonding, or ionic bridging with residual algal proteins [44,45]. This progressive mechanical reinforcement suggests that freshly extruded filaments can immediately support subsequent layers while continuing to strengthen for superior buildability and interlayer fusion. Simultaneously, the G″ roughly doubles from 1915 Pa to 3927 Pa, and tan δ rises gradually from 0.11 to 0.16, indicating a moderate increase in viscous dissipation. For DIW type extrusion-based 3D printing, the high initial G′ (>16 kPa) and low tan δ (0.11) ensure good shape retention and layer fusion right after extrusion. The moderate rise in G″ ensures that the material remains extrudable under nozzle shear yet rapidly recovers its structure post-deposition. Most importantly, the combination of strong G′ recovery and persistently low tan δ (<0.2) provides clear rheological evidence of highly efficient thixotropic self-healing [46]. Shear-induced disruption in the nozzle is rapidly and autonomously repaired within seconds to minutes via the reformation of reversible hydrogen bonds and physical entanglements, enabling printed layers to rebond seamlessly and allowing the final object to heal microcracks or printing defects simply by resting.

While these results clearly demonstrate rapid structural recovery and excellent short-term buildability following extrusion, they are inherently limited to the immediate post-deposition timescale. Importantly, the continuous increase in G′ observed during the 20 min time sweep already suggests that structural evolution persists beyond this window, indicating that the gel network remains dynamically reorganizing. However, the present rheological analysis does not capture longer-term retrogradation processes that occur over extended periods. Previous studies have shown that such retrogradation leads to increased starch crystallinity, further enhancing G′ but concurrently reducing network mesh size [12]. This indicates that the mechanical properties of *C. vulgaris* starch gels are likely to evolve significantly after printing, depending on storage time and conditions. Therefore, a systematic investigation into retrogradation-driven structural evolution and the long-term stability of printed constructs over days to weeks is warranted and will be addressed in future work. Table 3 provides a comparative overview of the rheological characteristics and 3D-printing relevance of various starch systems relative to the microalgal starch.

### 3.3. Structural Characterization

**Figure 7:**
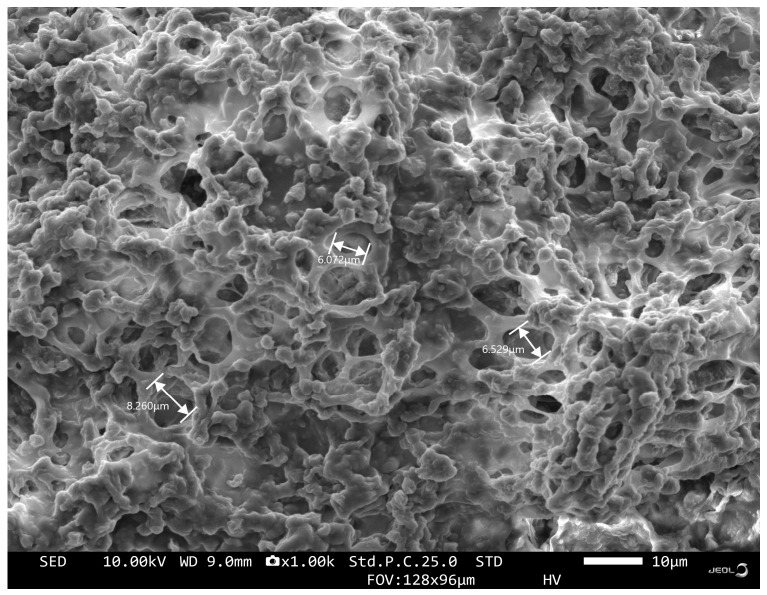
SEM image from cryofracture sample of *C. vulgaris* starch.

The SEM micrograph of the starch gel (Figure 7), obtained after liquid-nitrogen quenching and fracturing, displays a highly porous cryogel-like morphology with irregular cavities and a disrupted polymer framework. This structure does not represent the native hydrated gel but likely reflects the freeze-induced architecture formed as ice crystals displaced polymer chains and subsequent sublimation left solvent-derived voids. Despite this limitation, the persistence of a continuous skeletal framework after such extreme dehydration may indicate that the starch gel forms a cohesive and mechanically resilient polymer matrix. This qualitative structural robustness appears to align with the rheological behavior of the hydrated system, where the predominance of elastic moduli and the strong shear-thinning response likely arise from an interconnected network capable of supporting stable flow during extrusion and maintaining shape fidelity during layer deposition in 3D printing.

### 3.4. Implications for Extrusion-Based 3D Printing

The rheological profile of *C. vulgaris* starch aligns well with the requirements of DIW extrusion-based 3D printing, where controlled flow, shape fidelity, and rapid structural recovery govern successful layer-by-layer fabrication. The strong shear-thinning behavior enables easy flow through the nozzle under applied pressure, while the moderate yield stress ensures that deposited materials retain their shape without spreading. The predominance of elastic behavior (G′ > G″) across oscillatory tests further supports structural stability during layer deposition. Additionally, temperature-dependent strengthening of the gel network and the high gelatinization temperature provide a robust processing window, minimizing risks of premature thickening or nozzle blockage. Furthermore, the rapid structural recovery observed in the 3ITT test indicates that the material can quickly rebuild its internal network after extrusion, which is essential for maintaining material integrity and promoting interlayer adhesion.

In summary, *C. vulgaris* starch demonstrates promising suitability for DIW extrusion-based 3D printing. It forms a robust, physically cross-linked, elastic gel with a high G′≈13–24 kPa and a low tan δ < 0.2, showing good shear thinning, coupled with rapid thixotropic recovery, and an impressive thermal stability. These features allow for smooth extrusion, immediate shape retention, strong interlayer adhesion, and the creation of slump-free, self-supporting structures. Its bio-based, biodegradable, and renewable nature, in combination with favorable rheological properties, makes microalga starch one of the most promising alternatives for this application.

## 4. Conclusions

The steady and dynamic rheological properties of *Chlorella vulgaris* (*C. vulgaris*) microalga starch plasticized with 30% glycerin were investigated in this study to estimate its suitability for DIW bioplastic applications. The key findings are summarized as follows:

1. The steady shear rheological analysis demonstrates pseudoplastic (shear thinning) flow behavior with $\tau_{0}$ of 271.93 Pa, *K* of 59.47 Pa·s^n^, and *n* of 0.67.
2. The amplitude sweep clearly shows that the limit of LVE where the gel structure remains fully intact and undisturbed extends up to 0.6% strain, with G′ staying constant at ≈13,500 Pa and tan δ ≈ 0.1. This confirms good structural stability under small deformations typical of handling and low shear conditions.
3. The frequency sweep analysis performed in the LVE at 0.5% strain confirms a true strong physical gel with G′ remaining essentially frequency independent and significantly higher than G″ across the entire tested range (0.1–100 rad/s) and tan δ < 1. This demonstrates an outstanding shape stability and slump resistance at rest, effortless flow during extrusion, and rapid structural recovery after deposition.
4. The temperature sweep rheological analysis shows a predominantly elastic gel with G′ consistently surpassing G″ and no crossover point, exhibiting gradual increase in G′ (from 1.1 × 10^5^ Pa at 25–32 °C to a maximum of 6.6 × 10^5^ pa at 92 °C and η* (from 1.8 × 10^7^ mPa·s at 25–32 °C to a plateau around 1.06 × 10^8^ mPa·s above 90 °C, without thermal softening or melting. This is possibly due to granule swelling and amylose leaching; however, if algal proteins are present and interact with the matrix, they may also contribute to the observed reinforcement.
5. The starch exhibits promising thixotropic behavior, with viscosity dropping over six orders of magnitude under high shear (300 s^−1^) and recovering almost completely within seconds after shear removal, ensuring smooth extrusion and immediate shape retention.

The listed results therefore proved that *C. vulgaris* starch indeed possesses the ideal rheological characteristics for DIW extrusion-based 3D printing, such as a strong elastic gel structure, good shear-thinning behavior, fast thixotropic recovery, and thermal and mechanical stability. These promising rheological properties not only enable smooth extrusion, immediate shape retention, and layer fusion, but also make it an ideal material for high-resolution self-supportive 3D printing bioplastic. Overall, these findings position *C. vulgaris* starch as a promising microalgae-derived platform material for sustainable bioplastics, with potential applications in advanced additive manufacturing, biodegradable packaging, and other eco-friendly material systems. Future work should focus on conducting actual print trials to validate the rheological behavior in practice and evaluate the final bioplastic structures’ mechanical properties, surface finish, and dimensional accuracy.

## Figures and Tables

**Table 1:** Steady shear properties of starch gels from various botanical sources compared with *Chlorella vulgaris* (*C. vulgaris*) starch.

Starch	Wt.% of Starch	$\tau_{0}$ (Pa)	K (Pa·s)	*n*	$\eta_{{1s}^{-1}}$ (Pa·s)	Ref.
Rice	20	191	4000.4	−0.032	5000	[32]
Mashed potato + 2% potato starch	-	312.16	118.44	0.63	500	[33]
Corn	15	800	228.54	0.31	400	[7]
Cassava	13	-	560	0.25	1000	[34]
Wheat	13	-	159.6	0.09	1200	
Corn	13	-	283.8	0.12	1500	
Sweet potato	13	-	122.2	0.13	1000	
Potato	13	-	956.3	0.05	5800	
Buckwheat	13	-	834.6	0.03	7500	
Waxy corn	-	-	620	0.33	800	[35]
Potato	15	-	781.2	0.47	1000	[36]
*C. vulgaris*	20	271.93	59.47	0.67	400	This study

**Table 3:** Comparison of rheological properties and 3D-printing relevance of starch systems versus *C. vulgaris* starch.

Material	Key Rheological Findings	3D Printing Relevance	Comparison to Present Study	Ref.
Corn starch (CS) and rice starch (RS) gels	G′ increases with concentration and rise then fall with temperature; RS forms V-type crystals improving strength.	Identifies optimal printing windows: CS ≈ 20% at 70–75 °C; RS ≈ 15–20% at 75–8 °C	Agrees on temperature dependence of G′ (rise then fall); *Chlorella* shows higher gelation temp (92.7 °C)**,** implying a wider thermal processing window than CS/RS.	[47]
Corn starch variants (amylose 2%, 27%, 56%, 72%)	Higher amylose → higher G′, yield stress, viscosity; but excessive amylose reduces interlayer adhesion and extrusion continuity.	Optimal amylose (~27%) gave best printing accuracy (≈88%); high-amylose gels hard to extrude.	Notable alignment: *Chlorella* amylose ≈ 20.7%, close to the reported optimal range; this reinforces present study’s favorable balance between strength and extrudability.	[7]
Corn starch (20% suspension) treated at 65–100 °C	Leached amylose and short chains increase with T; G′ and shear recovery peak at intermediate T (CS-80), very high T → densification and difficult extrusion.	Demonstrates temperature-dependent optimum (CS-80) where network order and printability are maximized.	Direct parallel: both studies find a temperature optimum for G′ and recovery; *Chlorella*’s higher gelation temperature shifts that optimum upward, suggesting algal starch reaches peak network strength at higher processing temperatures.	[48]
Corn starch modified with glycyrrhizic acid (CS-GA)	Shear thinning; viscosity and G′ increase with GA content; bound water increases; improved thixotropic recovery.	High GA improved self-supporting and printing accuracy (best at 40% GA).	Consistent with present study on recovery and self-supporting: *Chlorella* shows strong recovery and high G′ without additives; CS-GA shows additive route to similar rheological gains.	[49]
Potato starch (PS) gels	G′ increase with concentration; with temperature G′ first increase then decrease; correlation with network compactness.	Optimal PS 15–25% at ~70 °C gave best printability (smooth extrusion + support).	Same trend with temperature (optimum then weakening at high T); *Chlorella’s* higher gelation temperature suggests better resistance to premature softening during hot-extrusion.	[50]

## Data Availability

The original contributions presented in this study are included in the article/Supplementary Material.

## Supplementary Materials

The following supporting information can be downloaded at: https://www.mdpi.com/article/10.3390/polym18121452/s1.
