# Supplementary figures and images for: Investigating Rheological Behavior of Chlorella vulgaris Starch: Implications for 3D Printable Bioplastic Material
Source: Polymers (Basel). 2026 Jun 10;18(12):1452. doi: 10.3390/polym18121452 (PMC13306264; doi:10.3390/polym18121452)

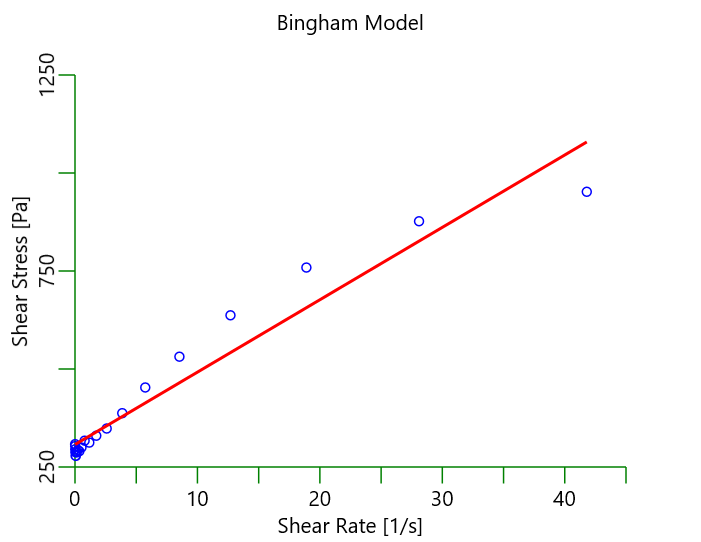

Supplement: Supplementary file 1 [file polymers-18-01452-s001.zip › Bingham.png]

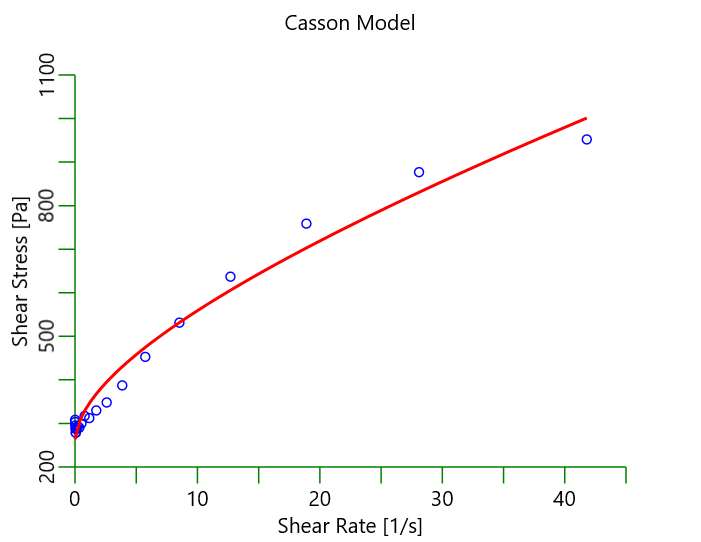

Supplement: Supplementary file 1 [file polymers-18-01452-s001.zip › Casson.png]

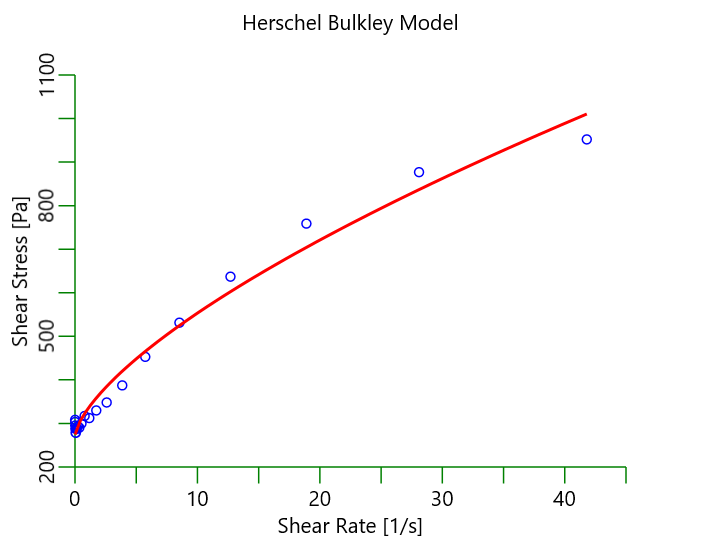

Supplement: Supplementary file 1 [file polymers-18-01452-s001.zip › Herschel Bulkley.png]

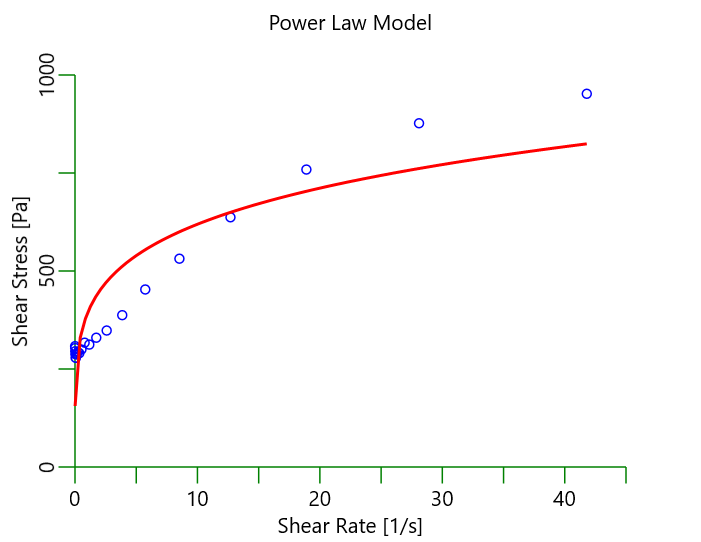

Supplement: Supplementary file 1 [file polymers-18-01452-s001.zip › Power Law.png]
